# Cisplatin-Membrane Interactions and Their Influence on Platinum Complexes Activity and Toxicity

**DOI:** 10.3389/fphys.2018.01898

**Published:** 2019-01-11

**Authors:** Nuno Martinho, Tânia C. B. Santos, Helena F. Florindo, Liana C. Silva

**Affiliations:** ^1^iMed.ULisboa – Research Institute for Medicines, Faculdade de Farmácia, Universidade de Lisboa, Lisbon, Portugal; ^2^Centro de Química-Física Molecular, Institute of Nanoscience and Nanotechnology and IBB-Institute for Bioengineering and Biosciences, Instituto Superior Técnico, Universidade de Lisboa, Lisbon, Portugal

**Keywords:** cisplatin mechanism of action, chemotherapeutic, membrane biophysical properties, membrane fluidity, membrane interactions, membrane permeability, sphingolipids

## Abstract

Cisplatin and other platinum(II) analogs are widely used in clinical practice as anti-cancer drugs for a wide range of tumors. The primary mechanism by which they exert their action is through the formation of adducts with genomic DNA. However, multiple cellular targets by platinum(II) complexes have been described. In particular, the early events occurring at the plasma membrane (PM), i.e., platinum-membrane interactions seem to be involved in the uptake, cytotoxicity and cell-resistance to cisplatin. In fact, PM influences signaling events, and cisplatin-induced changes on membrane organization and fluidity were shown to activate apoptotic pathways. This review critically discusses the sequence of events caused by lipid membrane-platinum interactions, with emphasis on the mechanisms that lead to changes in the biophysical properties of the membranes (e.g., fluidity and permeability), and how these correlate with sensitivity and resistance phenotypes of cells to platinum(II) complexes.

## Introduction

Cisplatin [*cis*-diamminedichloridoplatinum(II)] is a widely used chemotherapeutic drug in the treatment of various types of cancers, including testicular, bladder, ovarian, breast, head and neck, and small and non-small cell lung cancers. Despite its effectiveness, cisplatin still displays significant side effects and mechanisms of resistance by cancer cells are widely reported. Therefore, combination therapies and innovative cisplatin-based therapies have been pursued to improve its clinical use and overcome intrinsic acquired resistance. These range from the development of more effective and less toxic cisplatin analogs (e.g., carboplatin, oxaliplatin, and heptaplatin), to the use of drug carriers that deliver cisplatin in a more controlled way (Johnstone et al., 2016). For the latter, nanotechnology-based tools have been explored to modify the biodistribution profile of cisplatin by allowing a prolonged circulation time, improved accumulation at tumor site and subsequent internalization by cancer cells. This enhances its therapeutic efficacy, while preventing its off-targeted effects often associated to devastating adverse effects (Sun et al., 2014; Senapati et al., 2018). These nanosystems are particularly attractive to deliver combinations of drugs to a single targeted cell, which in fact constitutes the most frequent therapeutic schemes administered to cancer patients. In 2017, the liposomal formulation VYXEOS^®^(Celator Pharmaceuticals, Inc.) was approved for the treatment of certain types of newly diagnosed acute myeloid leukemia (Lancet et al., 2018). These liposomes co-entrap a combination of daunorubicin and cytarabine and constitute an important achievement by attesting the possibility of co-delivering distinct active agents with different pharmacokinetic profiles within a single carrier (Nikanjam et al., 2018).

Progress in this area is, however, dependent on the understanding of the mechanisms underlying cisplatin and other platinum(II) complexes action, toxicity and induced resistance. The primary target of cisplatin is the genomic DNA. Once inside the cell, cisplatin becomes activated and forms adducts with DNA, preventing further DNA replication that culminates in cell death (Dasari and Tchounwou, 2014). Moreover, the formation of these adducts is correlated with induced mutagenesis that contribute to the evolution of resistance response in tumor cells, and possible carcinogenesis that result in secondary malignancies often observed with some platinum(II) complexes (Sanderson et al., 1996; Szikriszt et al., 2016; Boot et al., 2018). Therefore, avoiding the formation of these resistance mechanisms and maintaining selective targeting to cancer tissues becomes an interesting approach to combinatorial therapy with nanosystems. Nonetheless, other mechanisms not dependent of DNA replication have been implicated in cisplatin cytotoxicity, including oxidative stress, modulation of calcium signaling and activation of various stress signaling cascades (reviewed in Dasari and Tchounwou, 2014).

**FIGURE 1:**
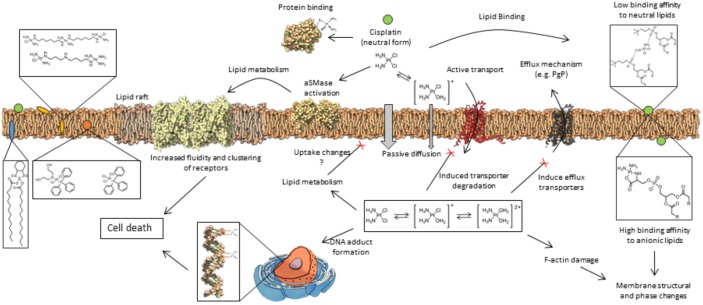
General mechanisms of interaction between platinum(II) complexes with cells. Different forms of platinum(II) complexes interact at different depths of the lipid membrane, which also influences their ability to permeate the cell membrane. Once inside the cell, platinum(II) complexes promote a cascade of events that lead to cell death or resistance mechanisms to platinum(II) complexes. These mechanisms are described in detail in the text.

Besides its effects at the intracellular level, it is now recognized that cisplatin-induced cytotoxic events might start at the level of the PM, where it directly interacts with proteins and lipids, causing alterations in membrane structure (Sun et al., 2014; Senapati et al., 2018) and biophysical properties (Lacour et al., 2004; Huang et al., 2010). Some of these events are summarized in Figure 1. Such alterations are likely to impact cell signaling events that result in cancer cell death. However, it may also have implications in the activation of mechanisms that lead to cancer cell resistance or those associated with cisplatin-side effects. In this review, we summarize the current knowledge on cisplatin-membrane interactions obtained from studies performed both in artificial membrane systems and living cells, and critically address how these interactions influence cell response to cisplatin.

## General Considerations on the Physicochemical Properties of Cisplatin and Analogs

**FIGURE 2:**
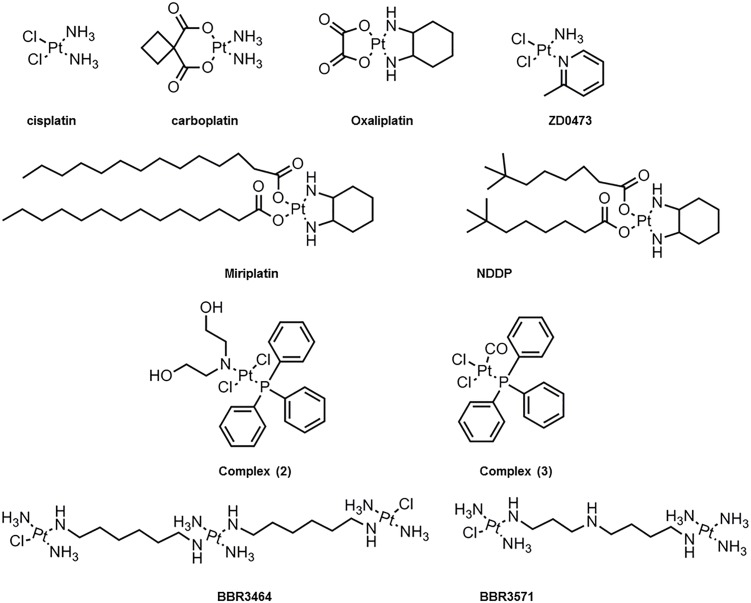
Molecular chemical structure of several platinum(II) compounds studied for interaction with lipid membranes.

**FIGURE 3:**
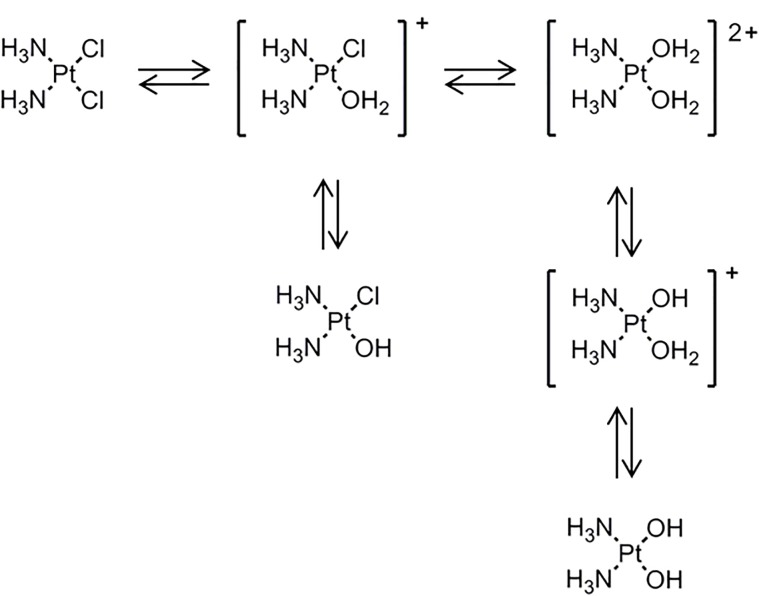
Cisplatin equilibrium in aqueous medium where it exchanges chloride with hydroxyls and water to form the so called aquated species. [Scheme was redrawn based on reference (Boot et al., 2018)].

Several platinum(II) complexes have been explored for their antitumor activity, commonly attributed to platination of nucleic acids. Among them, cisplatin has been the most studied but suffers from severe limiting side effects that have prompted second generation platinum(II) complexes. Cisplatin is a squared planar platinum(II) complex composed by a central metal atom coordinated with two chlorides and two ammonia molecules in a *cis* configuration (Figure 2). Even though the amines are relatively inert, the two chlorides are relatively labile ligands that are prone to nucleophilic substitution. In fact, cisplatin in solution exists as an equilibrium of different neutral or positively charged “aquated” species (Figure 3) (Speelmans et al., 1996; Maheswari et al., 2000). The equilibrium of these species is dependent on the pH, temperature and chloride concentration. It is generally accepted that in the blood, where a relatively high concentration of chloride is present, the equilibrium minimizes the formation of positively charged species. However, inside cells, where the chloride concentration is much lower, the formation of cationic species is promoted. Furthermore, cisplatin aquated form is much more reactive at forming coordinated intra- and inter-strand cross-links with DNA that cancerous cells cannot repair (Maheswari et al., 2000). Despite the higher reactivity, the low chloride concentration inside the cells is, however, not the limiting factor to cisplatin cytotoxicity or resistance behavior since platinum accumulation and DNA platination was found to be similar in different cell lines with varied concentrations of chloride (Jennerwein and Andrews, 1995).

Due to the inherent cytotoxicity problems associated with cisplatin, several analogs have been synthesized (Montaña and Batalla, 2009) and have been tested for clinical use based on the balance between binding to nucleic acids, stability, water solubility, acceptable levels of toxicity and increased spectrum of activity. These modifications have been generally carried by chelation with anionic groups (e.g., carboplatin and oxaliplatin) or alterations to the amine (e.g., oxaliplatin) as can be observed in Figure 2. Furthermore, other substantial modifications to platinum(II) complexes have been tested including the addition of aliphatic chains (e.g., miriplatin) (Liu et al., 2016) or using a *trans*-configuration and positive charge as in the case of BBR3464 and BBR3571 (Billecke et al., 2006; Liu et al., 2006). These changes in platinum(II) complexes ultimately result in variations in size and spatial configurations that affect their permeation, hydrolysis and reactivity rate, as well as impact on cells-resistance mechanisms (Abu-Surrah and Kettunen, 2006). Moreover, the different analogs form adducts with the DNA that are different from one another. For example, the formed adducts can be more hydrophobic or bulkier (e.g., oxaliplatin) and thus contribute to increased DNA synthesis inhibition. Overall these factors result in clinical differences as exemplified by carboplatin, which has shown lower kidney and nervous system toxicity compared to cisplatin (Kelland, 1993).

Besides DNA as their primary target, cisplatin and its analogs have multiple off targeting interactions. Off target effects were early evidenced in patients where cisplatin caused hemolysis (Getaz et al., 1980), and due to the mechanisms of resistance that arise after prolonged exposure. It has been proposed that the labile nature of platinum(II) complexes is responsible for the associated off target effects, where chemical transformations lead to a dynamic cascade of cellular events. In particular, cisplatin has high affinity for N and S donors. Thus, covalent platinum-S complexes with proteins are relatively stable and can only be reverted by other strong nucleophiles that would otherwise render an irreversible bond (Wang et al., 1996). Therefore, even before cisplatin reaches the cells membranes it encounters many obstacles. This is clearly observed by its high protein binding (>95%) and biological terminal half-life (60–90 h) (Deconti et al., 1973).

Upon reaching the cell, cisplatin and its analogs can also interact with a variety of biomolecules including membrane proteins, small molecules harbored at the surface of the bilayer, as well as, lipids. This interaction will affect its cellular uptake and efflux, and ultimately its bioactivity and toxicity. Hence, it is of uppermost importance to understand the fundamental interactions of platinum(II) complexes at biological interfaces, so that the mechanisms of toxicity can be understood, and novel complexes can be designed. In particular, there has been an effort to understand the mechanisms by which cisplatin enters cells since its uptake is not fully understood (Wang et al., 1996). Moreover, understanding the mechanisms leading to drug efflux and/or decreased intracellular accumulation is pivotal, since they determine drug efficacy and are associated to drug resistance mechanisms. Below we present a summary of the mechanisms involved in cisplatin uptake and efflux and the importance of cellular membranes for them.

## Brief Overview on Cellular Uptake and Resistance Mechanisms

The entry of cisplatin in cells is a complex process (Maheswari et al., 2000). Upon contact with the PM, cisplatin and its analogs need to cross the phospholipid bilayer to reach its primary intracellular target (Figure 1). In this regard, evidence suggests that the major mechanisms involved in cisplatin uptake are through passive diffusion and through facilitated transporters in particular organic cation transporters, such as, copper transporters (Ctr1) (Ishida et al., 2002). Transmembrane passive diffusion is a process that depends on both the size and hydrophilic/hydrophobic nature of the molecule. Cisplatin has a small size and no net charge and thus it is expected to enter via passive diffusion, even if low due to its hydrophilic nature. Indeed, studies in model membranes confirm the ability of cisplatin to cross the bilayer through passive diffusion (Hromas et al., 1987; Gately and Howell, 1993; Min et al., 2007). These results are further supported by molecular dynamics simulations (Yesylevskyy et al., 2015). Studies in cells also showed that cisplatin can be taken up by passive diffusion mechanisms since its accumulation by concentration gradient up to its saturation point was not the rate limiting factor (Hromas et al., 1987; Gately and Howell, 1993; Min et al., 2007). Moreover, cisplatin structural analogs did not inhibit platinum accumulation inside cells. In addition, when cell transporters were inhibited (e.g., by temperature) there was still intracellular platinum accumulation to a certain extent (Gale et al., 1973). Evidence further showed that cisplatin diffusion in DOPC membranes was much slower at low chloride concentrations (Eljack et al., 2014), further highlighting the importance of neutral charge of cisplatin in its diffusion. Finally, other platinum(II) complexes have showed higher diffusion rate due to their more hydrophobic nature (Ghezzi et al., 2004).

The permeation of drugs through the membrane also depends on its composition. Biological membranes are commonly perceived as entities displaying lateral organization into compositionally and functionally distinct domains. The biophysical properties of these domains are different and affect the interaction of drugs with membranes. It is thus not surprising that drug affinity for those domains can be different due to the packing density and free volume within those domains. The membrane fluidity has therefore a critical role in drug diffusion since the larger interior volume of membranes in the liquid-crystalline phase is more prone to accommodate molecules. On the other hand, the insertion of molecules into the lipid bilayer or the existence of specific interactions with lipids may cause changes in membrane structure and lateral organization. This can affect protein insertion and/or conformation within the membrane, which can be translated into changes in cell signaling and other membrane-associated cellular processes. In fact, a very recent study (in preprint) using molecular dynamics showed that cisplatin diffusion is dependent on membrane composition. It was observed that cisplatin had higher permeability in DOPC membranes compared to complex models of membrane. Moreover, varying the levels of DOPE and DOPS to mimic cancer cells resulted in decreased diffusion compared to asymmetric normal membranes (Rivel et al., 2018). In addition, a variety of external factors (e.g., pH, concentration of ions) can influence membrane composition and fluidity that might result in a higher or lower uptake of platinum(II) complexes. In fact, several studies have shown that cisplatin accumulation and passive permeation into the cells could be modulated by a number of different factors including pH, osmolality, temperature, Na^+^K^+^ ATPase, docosohexaenoic acid, digitonin, genistein, halenaquinone and ouabain (Loh et al., 1992; Gately and Howell, 1993; Baowei and Kui, 1995; Marverti and Andrews, 1996). Furthermore, the different platinum(II) complexes are expected to be metabolized differently, and the resultant metabolites will interact differently with the lipid bilayer. As an example, the metabolism of oxaliplatin results in the formation of products that have shown greater cellular uptake while being more toxic than the precursor (Luo et al., 1998, 1999; Graham et al., 2000).

There are also several resistance mechanisms associated with treatment of cisplatin that result in reduced binding of the drug to the DNA. These include the ability of cells to repair cisplatin-induced damage, as well as increase of pro-survival events that detoxify the platinum (e.g., increase production of molecules with sulfhydryl groups, such as, proteins, glutathione or metallothioneins) (Siddik, 2003; Galluzzi et al., 2014; Amable, 2016). Recently, the reduced intracellular accumulation of cisplatin has also been suggested to be a major factor for resistance. Cisplatin can trigger degradation of its transporter at the membrane interface resulting in lower influx rate (Holzer et al., 2006; Lin et al., 2002). Moreover, increased expression of MRP1 and MRP4 transporters results in reduced accumulation of cisplatin and oxaliplatin (Beretta et al., 2010). Similarly, TMEM205 protein was found to be overexpressed in resistant cells and when transfected into sensitive KB-3-1 cells it conferred resistance to cisplatin of approximately 2.5-fold (Shen et al., 2010). In addition, it was shown that the overall membrane permeability of resistant cells is lower compared to sensitive cells (Marverti and Andrews, 1996), thus resulting in a reduced intracellular cisplatin accumulation. However, whether this effect is due to changes in lipid composition is not fully understood (Liang et al., 2004), as it will be discussed below.

In summary, the composition and biophysical properties of cellular membranes significantly affects cisplatin uptake. In addition, several intrinsic and extrinsic factors may modulate the features of biological membranes and thus influence cisplatin-membrane interactions. Therefore, it is highly desirable to understand how these interactions occur prior to binding to DNA so that new improvements can be carried in future platinum(II) analogs and/or delivery systems.

## Cisplatin-Membrane Interactions

### Mechanistic Interaction of Cisplatin With Lipids

Despite the binding of cisplatin to phospholipids is in order of magnitude lower compared to proteins, it has been shown to still be relevant in cells (Wang et al., 1996). In this regard, both the type of lipids in the membrane and the surrounding aqueous environment influence cisplatin-membrane interactions. This is supported by studies showing that binding of cisplatin to LUV composed of neutral lipids (DOPC, DOPE, SM) was negligible but significant in the presence of anionic charged lipids, such as, DOPA, DOPS, DOPG, phosphatidylinositol and cardiolipin (Speelmans et al., 1996). The binding to these lipids was also found to be stronger in chloride free buffers. This suggests that the formation of aquated species of opposing charge is the factor determining the binding to anionic lipids (Speelmans et al., 1997). On the other hand, direct phase contrast and scanning electron microscopy observation of erythrocytes exposed to cisplatin showed evident changes in their shape, suggesting that cisplatin affect the erythrocyte membrane structure (Suwalsky et al., 2000). Electron microscopy studies in ovarian carcinoma cells further revealed that cisplatin establishes contact points with the PM, might form spike-like structures that connect with the PM, or form deposits that span through the PM (Beretta et al., 2002). In addition, fluorescence quenching studies of membrane proteins and quantification of free platinum measured in rat human cell membranes, also suggested binding of cisplatin to cell membranes (Baowei and Kui, 1995). Lipid-cisplatin specific interactions, particularly in the lipid chain, were also detected in cells using Raman-spectroscopy (Lu et al., 1995b; Batista de Carvalho et al., 2016; Marques et al., 2017). Altogether, these results indicate that cisplatin interacts with membrane lipids and may contribute to its action.

**FIGURE 4:**
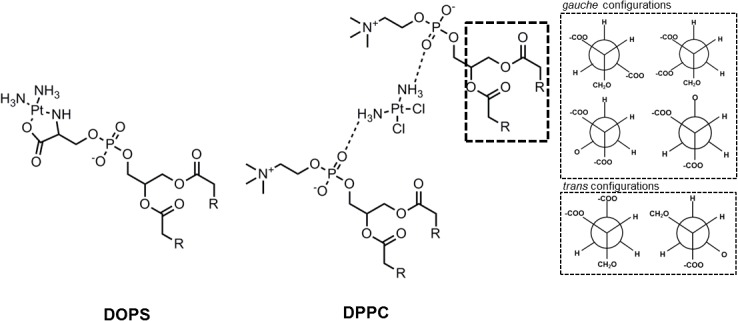
Interaction of cisplatin with DOPS and with two DPPC lipids. In the DPPC interaction it was observed further changes in the glycerol group that altered the common *gauche* configurations to a *trans* configuration. [Scheme was redrawn based on references (Suwalsky et al., 2000; Beretta et al., 2002)].

Mechanistic studies are therefore valuable to understand the interactions that result from binding (charge-charge interactions) or coordination to the head groups and whether these can lead to alterations in the features of the membranes. Examples are the studies that take advantage of artificial membranes. These allow identifying specific molecular interactions of cisplatin with lipids that can be used to correlate the cellular data. In this regard, differential scanning calorimetry (DSC) studies performed with DPPC LUV, showed that cisplatin altered the pre-transition of gel phase to ripple phase but not the main transition temperature of the gel to the fluid phase. This was attributed to rearrangement of the head groups with complexation of cisplatin to two phosphate headgroups, that did not cause alterations on the lipid chains (Figure 4) (Shen et al., 1991). Similar results were observed for the cationic aquated species of cisplatin in DPPC LUV, where strong alterations in the headgroups consistent with *gauche* to *trans* transformation in the glycerol moiety were observed (Wang et al., 1991, 1996). Additionally, these changes recovered slowly, in a process that started from the headgroups and extended to the interior of the membrane, as measured by NMR and infrared (IR) spectroscopy (Lu et al., 1995b; Wang et al., 1996). However, fluorescence spectroscopy studies showed that despite the conformational changes induced by cisplatin on the lipid bilayer, no significant changes in the fluidity of the membrane were observed. In fact, both the fluorescence anisotropy of DPH, which reports the lateral and rotational mobility of the probe, and the GP of Laurdan, which provides information on the dipolar relaxation of the probe and that in membranes is related to the hydration of the bilayer, remained unchanged upon interaction of cisplatin with the membranes. However, these studies were performed in DMPC LUV (Suwalsky et al., 2000), and the effects of cisplatin and its analogs in the fluidity of DPPC or other lipid component membranes might be different. Indeed, in liposomes mixtures of DOPC:DOPS (1:1) it was observed by atomic force spectroscopy (AFM) that the incorporation of cisplatin resulted in stiffer membranes compared to vesicles with no cisplatin (Ramachandran et al., 2006). Moreover, ^31^P NMR measurements of bilayers formed from pig lipid extracts, showed that cisplatin caused changes in the phase behavior of the membranes, which were consistent with the co-existence of at least 3 lipid phases, including a non-lamellar hexagonal II phase (Lu et al., 1995b; Fang et al., 2000). However, these alterations were not observed in model LUV composed of a mixture of phosphatidylcholine/cholesterol/PEG-DSPE (51:44:5) (Peleg-shulman et al., 2001), showing the specificity of membrane lipid composition in cisplatin-mediated effects.

Cisplatin interaction with the serine group of phospholipids (e.g., PS) has also been extensively studied. Using electron microscopy and X-ray diffraction, it was observed that cisplatin inserted into the inner monolayer of human erythrocytes and induced stomatocytic shape changes (Suwalsky et al., 2000), most likely due to the formation of cisplatin-PS complex (Burger et al., 1999; Suwalsky et al., 2000). PS is a negatively charged phospholipid that is mostly located in the inner leaflet of the PM of non-cancer cells. This lipid is crucial in many cellular events as it interacts with proteins anchoring them to the inner monolayer of the PM (Buckland and Wilton, 2000). However, cancer cells present an increased level of PS in the external leaflet of the PM (Sharma and Kanwar, 2017; De et al., 2018), which might promote changes on its surface charge and lipid packing. These changes in the biophysical properties of the PM might alter cell functioning and response to chemotherapy. In addition, PS influences the activity of enzymes including the Na^+^/K^+^-ATPase (Schuurmans Stekhoven et al., 1992) a transporter that has been reported to be involved in the uptake of cisplatin (Andrews et al., 1991; Gately and Howell, 1993).

Solid-state NMR studies further suggest that cationic aquated cisplatin establishes an electrostatic interaction with PS, causing its reorganization (Jensen et al., 2010). This results in changes in the lipid bilayer, including the formation of lamellar phases with increased phospholipid head group/phosphate mobility in the bilayer (Jensen et al., 2010). Other studies showed that cisplatin induces structural perturbations in model membranes containing DAPS, but not in lipid membranes composed of zwitterionic lipids (Suwalsky et al., 2000). Moreover, and contrary to what has been described for cisplatin-DPPC interaction, the phosphate group is not responsible for platinum coordination to POPS but rather it formed a complex with both the carboxyl and amine group of the serine headgroup (Figure 3) that was stabilized by the phosphate group (Speelmans et al., 1997). These observations show that cisplatin-membrane interactions are dependent on lipid headgroup structure. The headgroups of lipids determine the hydrophilic/hydrophobic balance at the surface of the bilayer and have major influence on the conformation, transition from gel to liquid crystalline phase, hydration levels, as well as, penetration of water, ions and small molecules (Serrallach et al., 1983; Scherer, 1989). Even small alterations in the headgroup of phospholipids, such as, addition of an extra methyl in the glycerol moiety results in conformational changes of lipid bilayers (Lewis et al., 1986), that can influence drug-membrane interactions. Likewise, coordination of cisplatin to serine groups of phospholipids is expected to result in changes in membrane structure to accommodate the platinum complex. It should, however, be stressed that platinum-PS complex formation is not always observed. In fact, the platinum-PS complex has not been detected in some cancer cells and in intact erythrocytes (Burger et al., 1999; Shirazi et al., 2003). This has been attributed to the fact that cisplatin can react with other cellular components and not be available to coordinate with PS. Moreover, the membrane permeability was shown to be an important factor for the formation of platinum-PS complex. Indeed, in aged erythrocytes and in cells exposed to digitonin where the membrane permeability was increased, the platinum-PS complex was detected (Burger et al., 1999).

Further insight into the interactions of platinum(II) compounds with lipid bilayers was obtained from other cisplatin analogs (Figure 2). As expected, the positively charged BBR3464 and BBR3571 exhibited higher levels of cellular uptake compared to cisplatin due to their enhanced hydrophobic nature. In addition, DSC and NMR studies showed that these compounds interact not only with the phosphate groups but also with the core of the bilayer (Billecke et al., 2006). These analogs were shown to coordinate with DPPG, DPPA, and DPPS and cause changes in the pre-transition temperature of these lipids, but not of DPPE and DPPC. These interactions occurred rapidly within 20 min likely due to electrostatic and hydrogen interactions, followed by slower formation of platinum-lipid complexes as observed by ^31^P NMR (Liu et al., 2006). Other analogs [Figure 2, complex (2) and complex (3)] were shown to cause phase changes consistent with hexagonal phase formation upon interaction with DMPC membranes as measured by NMR (Nierzwicki et al., 2014). The effect of structural modifications of the platinum complexes on their interactions with lipid membranes was further explored by comparing complex (2) and (3) with cisplatin. While cisplatin induced significant ordering of the alkyl chains with decrease in the area per lipid molecule and membrane elasticity, complex (2) only showed a small effect in the ordering (Nierzwicki et al., 2014). Moreover, molecular dynamics of these systems showed that both complex (2) and (3) partitioned to the interior of the membrane whereas cisplatin accumulated at the surface (Nierzwicki et al., 2014). It can therefore be concluded that the mechanisms underlying cisplatin- and analogs-membrane interactions depend both on the platinum(II) complex structure (particularly hydrophobic moieties) and membrane lipid composition. This interplay will influence the type of interactions (e.g., electrostatic, covalent, etc.) and the depth at which these occur. Moreover, they will influence the organization, structure and properties of the membrane. Thus, it is fundamental that future studies integrate analysis of lipid interactions as a strategy to improve the toxicity profile of cisplatin.

The membrane fluidity is determined by its lipid composition and the interactions established between lipids and proteins, which might be affected by interaction with external agents. In this regard, EPR spin-labeling studies showed that several platinum(II) complexes increase the transition temperature of human erythrocytes membrane, resulting in 1.4–3.5% increase in lipid membranes order (Wang et al., 1996). Moreover, fluorescence photobleaching studies in ascites cancer cells revealed that the lateral diffusion of phospholipids was also lower after treatment with a platinum(II) complex (Wang et al., 1996), consistent with a cisplatin-induced decrease in membrane fluidity. The type and amount of aquated species, as well as, the presence of anionic lipids appeared to be responsible for this effect. Indeed, DSC measurements showed that platinum(II) complexes were able to increase the *T*_m_ of anionic (DPPG) but not of zwitterionic (DPPC) phospholipids, indicating that the charge of the lipid head group also influences the interaction and the resultant cisplatin-induced effects. Such electrostatic interactions established between platinum(II) and phospholipids are likely to be responsible for the more rigid and less fluid membrane (Speelmans et al., 1996). This is further supported by fluorescence studies showing that the influence of ions in the aqueous environment (e.g., Ca^2+^, Mn^2+^, Mg^2+^. Cu^2+^, and Zn^2^) altered the fluidity of the membrane by interacting with phosphates (e.g., electrostatic complexation), which in turn resulted in higher encapsulation of cisplatin into LUV (Liang and Huang, 2002; Liu et al., 2015). These results seem to indicate a connection between the ability of platinum (II) complexes to establish electrostatic interactions in model membranes and decreased membrane fluidity induced by complexation with the headgroups. However, in biological membranes this interaction becomes much more complex, as it will be discussed in the next section.

Besides changes in membrane fluidity, it was also reported that cisplatin might create small conducting defects in the inner hydrocarbon core. Such defects increase ion passage, as shown by ion conducting studies in a phospholipid mix of egg yolk in presence of cisplatin (Maheswari et al., 2000). These results also correlated well with *in vitro* studies in epithelial cells where a drop of 89% on the TEER (Trans Epithelial Electrical Resistance) was observed 24 h upon addition of cisplatin to the basolateral side of C7 cells (Ekle and Chwerdt, 2004).

Overall literature data highlight that cisplatin interacts with lipid bilayers but its effect are complex and dependent on a variety of extrinsic factors, including pH and chloride concentration, as well as, membrane composition and other unknown factors (Mann et al., 1990; Lu et al., 1995a; Liang and Huang, 2002; Jensen and Nerdal, 2008). In fact, the importance of the physical state of membranes for the mechanisms of cisplatin and other platinum(II) complexes interaction remains to be determined. It is likely that differences in membrane composition can lead to structural differences that in turn affect the permeability of cisplatin. Ultimately, understanding how drug-membrane interactions occur creates new opportunities to develop innovative cisplatin-based therapeutic strategies. In particular, tracking issues related to the bypass of mechanisms of drug resistance originated by efflux or the repair mechanism can further guide the development of newly improved platinum(II) complexes or DDS.

### Cell Signaling Modulation by Cisplatin Interaction With Cell Membrane

Cell signaling events are often dependent on membrane organization and structure. For instance, activation of apoptotic cascades has been related to changes on membrane biophysical properties fundamental for receptor clustering (Grassmé et al., 2001a). Since cisplatin can induce alterations in the fluidity of the membrane it is not unlikely that this drug might modulate signaling events upon its interaction with the membrane. In fact, relevant therapeutic concentration of cisplatin was shown to cause a transient increase in membrane fluidity of HT29 cells that persisted for 4 h as measured by EPR of 12-DSA (Lacour et al., 2004). These changes on membrane fluidity were accompanied by the formation of large CD95 aggregates and the redistribution of CD95, together with the DISC-forming molecules FADD (Fas-Associated protein with death domain FasL) and procaspase-8 into PM lipid rafts (Lacour et al., 2004; Rebillard et al., 2007). The CD95 aggregates were found to be promoted by a rapid and transient activation of aSMase (Lacour et al., 2004), which occurred in response to a decrease in intracellular pH mediated by cisplatin via inhibition of NHE1 (Rebillard et al., 2007; Shirmanova et al., 2017). Interestingly, the activation of this signaling pathway correlated with the formation of ceramides that occur at the external leaflet of the PM due to aSMase-mediated SM hydrolysis (Lacour et al., 2004; Rebillard et al., 2007; Zeidan et al., 2008). This is supported by previous studies showing that ceramide domains are important for the clustering of CD95 at the cell surface and subsequent amplification of CD95 signaling (Grassmé et al., 2001a,b; Lacour et al., 2004; Rebillard et al., 2007). Increased aSMase activity with concomitant upregulation of FAS was also recently described (Maurmann et al., 2015) in ovarian carcinoma A2780 cell line, suggesting a common mode of action for cisplatin when used in the treatment of different cancer cells. It should, however, be stressed that the observed increase in membrane fluidity induced by cisplatin is biophysically not compatible with the formation of ceramide enriched domains since these are expected to be highly ordered (Castro et al., 2014; Pinto et al., 2014) and therefore cause a decrease in membrane fluidity, as already reported for live cells (Pinto et al., 2014). Therefore, changes in membrane fluidity observed in these cells upon treatment with cisplatin might derive from other factors, not related to ceramide formation.

Other studies also taking advantage of EPR suggested that cisplatin-induced membrane fluidification was implicated in activation of Fas death receptor pathway that in turn resulted in rapid and transient re-organization of F-actin microfilaments (Rebillard et al., 2010). This is in agreement with the observation that cisplatin-induced damage to F-actin in proximal tubular cells occurred prior to changes in nuclear morphology (Kruidering et al., 1998). Furthermore, F-actin damage may cause an increased monolayer permeability (Kroshian et al., 1994). These observations are also in accordance with a work showing that cisplatin is able to induce permeabilization of the PM of T24 cells (Speelmans et al., 1996, 1997; Zhang et al., 2015). The importance of cisplatin interaction with actin was further shown by AFM in human ovarian cancer cells where F-actin was indeed considerably remodeled by cisplatin, causing changes in cellular nano-mechanics and increasing cell stiffness determined (Sharma et al., 2012). This is of major importance since the polymerization of actin monomer (G-actin) and depolymerization of filamentous actin (F-actin) represent fundamental molecular processes critically involved in cell motility, morphology, transport, cytokinesis and intracellular signaling (Khaitlina, 2014; Rajakylä and Vartiainen, 2014). Therefore, cisplatin interference with actin might impair these activities increasing the toxic effects induced at the PM level.

However, literature data is controversial regarding the mechanisms by which cisplatin modulates cellular structural features. In this regard, PS120 fibroblasts observed by scanning electron microscopy presented changes on membrane morphologic features with the appearance of noticeable villosities and long protrusions after cisplatin treatment but no changes were observed in cell stiffness or actin cytoskeleton structure as measured by AFM (Milosavljevic et al., 2010). This suggests that cisplatin has a direct effect on the bilayer and not on actin. It should, however, be stressed that different studies use different cell lines (Kruidering et al., 1998; Milosavljevic et al., 2010; Sharma et al., 2012), different cisplatin concentrations (Kruidering et al., 1998; Milosavljevic et al., 2010) and different methods of analysis (Kruidering et al., 1998; Milosavljevic et al., 2010) that might all contribute to the observed differences. In fact, it was already shown that the time required for cisplatin-induced cytoskeleton remodeling and loss of F-actin was concentration dependent (Kruidering et al., 1998).

Altogether, these studies suggest that cisplatin-induced changes on membrane biophysical properties and cytoskeleton structure might underlie the clustering of death receptors and activation of apoptotic cascades. These events occur upstream of platinum-DNA adduct formation and are likely to be the early steps contributing to cisplatin cytotoxicity.

### Resistance to Cisplatin Induced by Changes on Membrane Biophysical Properties and Sphingolipid Metabolism

A large fraction of human malignancies rapidly becomes (or intrinsically is) insensitive to the cytotoxic effects of cisplatin (Siddik, 2003; Galluzzi et al., 2014). Many studies have tried to elucidate the process responsible for the resistance to cisplatin treatment to overcome it and improve the success of the therapeutic regimen (Siddik, 2003; Galluzzi et al., 2014). The complexity of the multiple factors involved is, however, a major obstacle for the development of an effective strategy to overcome resistance. Besides, all the proposed targets are non-specific for cancer cells, which further increase this challenge.

Conversely, different studies suggest that the biophysical properties of the PM of cancer cells, including membrane fluidity, are different from the ones of their non-cancerous counterpart (Deliconstantinos, 1987; Zoellner et al., 2015). Since cisplatin cytotoxicity starts at the PM level, these differences in membrane properties can be used as an advantage to develop a specific and directed therapy targeting the PM of cancer cells but also to improve the efficacy of cisplatin action. To that end it is necessary to identify biophysical features of different cancer cells, but also to understand how changes in their biophysical properties might correlate with the development of mechanisms of resistance to chemotherapeutics. In this regard, many studies suggest that cancer cells sensitive to cisplatin have an overall higher membrane fluidity compared to resistant cells. In addition, cisplatin seems to cause stronger changes in the biophysical properties of sensitive cells, suggesting that the early steps of cisplatin action might depend on biophysical mechanisms. However, literature is still controversial regarding this issue, as discussed below.

Atomic force spectroscopy studies suggest that cisplatin-resistant ovarian cancer cell lines present a significantly higher cell stiffness compared to their cisplatin-sensitive counterparts (Sharma et al., 2012, 2014). Furthermore, an increase in sensitive cells stiffness was observed after cisplatin treatment, whereas no alterations were observed for resistant cells (Sharma et al., 2012). This is also in agreement with the studies showing that cisplatin-induced changes in the transition temperature of the membranes were directly correlated with the degree of cisplatin resistance. Indeed, no significant changes on membrane fluidity were observed when resistant cells were treated with cisplatin compared to cisplatin-sensitive cells or cells with intermediate sensitivity to cisplatin, as observed in differences in the fluorescence intensity of merocyanine 540 between these cells (Liang and Huang, 2002; Raghunathan et al., 2015), or in differences in the ΔT*m* obtained by quantification through fluorescence microscopy of the fraction of giant PM vesicles formed from these cells containing coexisting liquid phases (Liang and Huang, 2001). Furthermore, modulating the fluidity of the membrane also resulted in different response to cisplatin. In models of cells of intermediary sensitivity to cisplatin, increasing the fluidity of the membrane with isopropanol resulted in an additive effect of increased fluidity upon addition of cisplatin. On the contrary, decreasing the fluidity of these cells with menthol, canceled the effect of increased fluidity by addition of cisplatin (Raghunathan et al., 2015). This result also correlated with the toxicity of cisplatin on those cells. When isopropanol was added to those cells, cisplatin treatment resulted in more cell death compared to cisplatin treatment alone. On the contrary, adding menthol to those cells resulted in lower toxicity by cisplatin (Raghunathan et al., 2015).

Studies performed with the human lung adenocarcinoma cell line A549 resistant to cisplatin (A549/DDP) also showed that these cells present a less fluid membrane compared to A549 cells sensitive to cisplatin (Liang and Huang, 2001, 2002). The differences of membrane fluidity were mainly located on the surface and middle layer of PM as measured by polarization of DPH (Liang and Huang, 2001, 2002). Lipidomic analysis of these cells showed that A549/DDP cells present a higher percentage of saturated fatty acids than A549 cells (Liang and Huang, 2001) with a higher level of C16:0 fatty acids and a decrease in C18:0 species (Liang and Huang, 2002). No difference in cholesterol concentration was found in the membranes between the two cell lines (Liang and Huang, 2002). The observed differences in membrane fluidity between these two cell lines can be explained by the different compositions and properties of the fatty acids present in these cells, where enrichment in lipids with saturated acyl chains cause a decrease in the fluidity and lateral diffusion of the membrane (Pinto et al., 2011). Alterations in membrane structure can impair receptors clustering and intracellular signaling that could lead to a decreased apoptotic response to cisplatin in A549/DDP cells. The passive diffusion and intracellular accumulation of cisplatin could also be reduced by an increase in lipid membrane packing, limiting its cytotoxic effect. This mechanism has been suggested for the confluence-dependent resistance phenomenon where the sensitivity of human colon cancer cells to cisplatin decreased with increasing cell-culture density (Dimanche-Boitrel et al., 1992). Fluorescence measurements of TMA-DPH polarization suggested that the altered drug penetration was due to a decrease in PM fluidity induced by cell confluence (Dimanche-Boitrel et al., 1992). It should also be mention that cisplatin causes an increase in membrane fluidity of A549 sensitive cells, having the opposite effect in A549/DDP resistant cells. These differential effects were associated to changes in phospholipid components between the PM of these two cell lines (Huang et al., 2003).

Cisplatin-resistant breast cancer MCF-7/S cells also have a different lipid profile compared to MCF-7/CP cells with increased content of cholesterol, SM, PG and PS and decreased levels of PC and PE (Todor et al., 2012). An increase in cholesterol and SM with a decrease in the PC/SM ratio strongly suggests a decrease in membrane fluidity of resistant cells as observed for A549/DDP cells (Todor et al., 2012).

In addition, cisplatin resistant ovarian cancer cells were shown to present a highly organized actin architecture with a more robust actin cytoskeleton and stress fibers compared to cisplatin sensitive cells (Sharma et al., 2012, 2014). A highly dense F-actin network could thus create a barrier preventing cisplatin uptake and conferring resistance or inefficacy of the drug, for instance by influencing cell membrane biophysical properties, including membrane fluidity (Sharma et al., 2012, 2014). In contrast, human epidermal KCP-20 carcinoma cells resistant to cisplatin present more fluid PM than the sensitive KB-3-1 cells (Liang et al., 2004). Nevertheless, the increased fluidity observed in KCP-20 cells PM may not be responsible for cisplatin resistance, since the membrane potential of KCP-20 cells was found to be hyperpolarized compared to the low level resistant KB-CP.5 and sensitive KB-3-1 cell line. The differences in membrane potential were accompanied by increased expression of K^+^ channels on the PM of KCP-20 cells (Liang et al., 2004) that can contribute to the resistance mechanisms.

Finally, as mentioned above, it has been described that cisplatin cytotoxic mechanism comprises the activation of aSMase (Lacour et al., 2004; Rebillard et al., 2007; Maurmann et al., 2015). However, the activation of this pathway also seems to be dependent on cell sensitivity to cisplatin (Maurmann et al., 2015). Accordingly, while aSMase pathway is activated in ovarian carcinoma A2780 cell line the same is not observed for the cisplatin-resistant counterpart A2780/C30 cell line (Maurmann et al., 2015). Even 24 h after treatment no alterations were observed in the levels of FAS, FASL, BCL2, CASPASE-3 and -9 transcripts corroborating the resistant state of the cells to cisplatin (Maurmann et al., 2015). These results evidence the importance of aSMase activation and consequent changes on PM biophysical properties in the mechanism underlying cisplatin-induced cell death. Moreover, it also highlights that a dysregulation of sphingolipids metabolism on resistant cancer cells can be implicated in the development of such resistance, as already describe for other therapeutic regimens (Giussani et al., 2014).

In fact, sphingolipids are ubiquitous components of eukaryotic cell membranes known to be involved in a variety of cellular processes including proliferation, growth, differentiation, apoptosis and membrane structure (Futerman and Hannun, 2004; Lahiri and Futerman, 2007; Carreira et al., 2015). Dysregulation of their metabolism is evident in various pathological conditions including cancer (Gulbins and Petrache, 2013; Don et al., 2014; Giussani et al., 2014; Roh et al., 2016). Formation of ceramide in response to a variety of stimuli is typically a hallmark for the activation of cell death pathways (Hannun and Obeid, 2008; Saddoughi and Ogretmen, 2013). Accordingly, it is not surprising that cells treated with cisplatin show elevation in ceramide levels. Indeed, elevation in C16-, C18-, and C-20 ceramide species was observed in BKM cells sensitive to cisplatin (Siskind et al., 2010). However, no changes in ceramide levels were observed in DKO cisplatin-resistant cells (Siskind et al., 2010), suggesting that these cells developed mechanisms to prevent ceramide-induced cell death. In addition to changes in the ceramide pathway, cisplatin-resistant cells tend to overexpress glucosylceramide synthase (Roh et al., 2015; Tyler et al., 2015), and have higher levels of glycosphingolipids with longer carbohydrate chains and α-hydroxy fatty acids (Kiguchi et al., 2006). The distribution of the α-hydroxy groups of fatty acids in the hydrogen bonding region of the lipid bilayer, will influence membrane structure and interaction with intrinsic proteins (Kiguchi et al., 2006). These changes might contribute to impairment in signaling cascades including those leading to cell death. Such observations placed sphingolipid metabolism in the spotlight, as one of the target pathways for the development of therapeutic strategies to treat cancer disease. Accordingly, studies using different cancer cell lines have already shown that modulating the level of certain sphingolipids and specific enzymes from sphingolipid metabolism is a good strategy to increase cells sensitivity to cisplatin (Min et al., 2005, 2007; Roh et al., 2015, 2016; Sassa et al., 2012), but also to other anticancer drugs (Charles et al., 2001; Chalfant et al., 2002; Dumitru et al., 2009; Martínez et al., 2009).

As suggested above, the observed differences on the biophysical properties of the membrane and actin structure of cisplatin-resistant cells could be the direct cause of resistance due to changes on membrane structure and consequent impairment of receptors clustering and intracellular signaling that could lead to a decreased apoptotic response to cisplatin in resistant cells. The passive diffusion and intracellular accumulation of cisplatin could also be reduced by an increase in lipid membrane packing, limiting its cytotoxic effect as observed for the confluence-dependent resistance phenomenon (Dimanche-Boitrel et al., 1992). However, these differences could also have an indirect contribution by promoting changes on the structure and/or function of transmembrane proteins, impairing their functioning and, altering their binding and/or response to cisplatin. For instance, it is known that *P*-glycoprotein has greater affinity to substrates when the lipid bilayer is in the gel phase than in the fluid phase (Clay and Sharom, 2013), suggesting that increased lipid packing, as observed in resistant cancer cells, might enhance *P*-glycoprotein-mediated cisplatin efflux. Moreover, protein kinase C migrates to the PM when activated by cisplatin (Muscella et al., 2015). Alterations on membrane composition and properties could alter protein kinase C binding to the PM and hence its activity and cell sensitivity to cisplatin action. Finally, cisplatin destabilizes membrane anchoring of actin filaments, leading to rearrangement of filamentous actin network and overall loss of cellular processes (Zeidan et al., 2008).

Overall, the evidence presented herein suggests that sphingolipid metabolism and membrane biophysical properties are two interconnected factors associated to cisplatin-induced cell death and/or development of cisplatin-resistance mechanisms.

## Innovative Cisplatin-Based Therapeutic Strategies

There has been a tremendous effort in designing new platinum(II) based drugs or to develop and improve new delivery strategies to target tumors. Because platinum(II) complex have inherent clinical problems, it has been extensively studied the use of DDS to modulate their toxicity. There have been several types of DDS tested which generally are used to circumvent solubility problems, modulate release rates, avoid resistance mechanism and avoid non-tumor tissues (Howell and Fan, 2010; Kim et al., 2009). This type of approach has been extensively tested and several DDS are under clinical development and are outside of the scope of this review.

Of note is the Nanoplatin^TM^ developed by Kazunori Kataoka, which constitute polymeric micelles conjugating cisplatin derivatives (NC6004) (reviewed in Mochida et al., 2017). Different clinical trials are being conducted using NC6004 in combination with other active agents, such as gemcitabine, 5-Fluorouracil and cetuximab (ClinicalTrials.gov, 2014, 2016a,b, 2018b,c).

Moreover, honorable mentions must be given to liposomes since they constitute the majority of the nanosystems already approved for clinical use and, in addition, are a tool that provide interesting information regarding platinum(II) complexes interaction with lipids. Liposomes have been commonly used for platinum formulations such as SPI-077 and Lipoplatin for cisplatin and Lipoxal for oxaliplatin (Hamelers et al., 2006). These lipidic nanoparticles protect cisplatin from the outside biomolecules that would normally react with platinum(II) complexes and then fuse with cells releasing its contents. Interestingly, cisplatin in a lipid suspension of multi lamellar vesicles (MLVs) with equimolar amounts of dioleoyl-PS and dioleoyl-PC showed to be more cytotoxic than cisplatin or cisplatin mixed with the lipids (Burger et al., 2002).

Another interesting case from the use of liposomes has been the combination with NDDP [Bis-neodecanoato-1,2-diaminocyclohexaneplatinum(II)] and miriplatin (Figure 2). In particular, miriplatin is a phospholipid dicarboxylic acid platinum complex that has been developed for the hepatocellular carcinoma. Both these complexes are highly insoluble and thus liposomes offer an alternative for their use. However, in the case of NDDP the lipid composition of the liposome was shown to determine its biological activity and toxicity. In fact, using liposomes composed of DMPC, DMPG:DMPC (7:3 and 3:7) as well as DMPG, it was observed that the presence of DMPG was essential for the NDDP activity. These studies demonstrated that NDDP was inactive, and the reaction intermediate between DMPG and NDDP was the active product (Orena et al., 1991; Perez-Soler et al., 1991; Perez-soler and Khokhar, 1992; Liu et al., 2016). Interestingly, even though miriplatin has similar structure to NDDP, it is active on its own and does not require DMPG. Nevertheless, the loading capacity of different liposomes to miriplatin was shown to be higher for DMPC, DMPG and DPPC in comparison to HSPC, DPPG, and DSPG (Liu et al., 2016).

Also worth mentioning are the DDS that take advantage of sphingolipids (Webb et al., 1995; Mehta et al., 2000; Shabbits and Mayer, 2003; Semple et al., 2005; Wang et al., 2017) or modulate sphingolipid metabolism (Lucci et al., 1999), as a synergistic strategy to enhance chemotherapeutics-induced cell death. Examples of such strategies include reports of DDS that combine both the effect of the encapsulated anti-cancer drug with the effects of SM or ceramide to increase the intracellular levels of ceramide and enhance the apoptosis, even in resistant cell lines. In general, the use of liposomes in this strategy has shown significant transport of the drug across the PM with enhanced accumulation of ceramide levels that lead to increased sensitivity to the cytotoxic effects of the drug (Webb et al., 1995; Mehta et al., 2000; Shabbits and Mayer, 2003; Semple et al., 2005; Wang et al., 2017). However, to the best of our knowledge, the combination with sphingolipid-based DDS has not been studied for platinum(II) based drugs and thus further research is needed.

Interestingly, there are growing evidences that cisplatin by itself has immune modulatory effects, which includes an improved antigen presentation through the Major Histocompatibility Complex (MHC) class I molecules, increased infiltration and proliferation of effector immune cells and their lytic activity, and decreased recruitment of immune suppressive cells, such as regulatory T (Treg) cells and Myeloid-derived Suppressive cells (MDSCs) (reviewed in de Biasi et al., 2014; Hato et al., 2014). As a result, considerable efforts have been recently devised to assess the synergy between cisplatin and several emerging immune therapeutic approaches. Many of them are under clinical development, such as the combination of cisplatin with dendritic cell vaccination (NCT02285413), with gemcitabine and anti-PD-L1 antibody Avelumab (CT03317496), or with anti-PD-1 Nivolumab (NCT03294304) and anti-CTLA-4 Ipilimumab (NCT03520491, NCT03101566) (ClinicalTrials.gov, 2018a).

Besides being clear that cisplatin may have an additional positive effect by modulating host immune system, the mechanisms underlying the interaction of cisplatin with the different subsets of immune cells within tumor microenvironment still needs to be clarified.

## Conclusion

The clinical use of platinum(II) complexes and, in particular, cisplatin has been limited due to side effects and resistance mechanisms that arise on continuous treatment. These effects are not limited to changes in the primary target of cisplatin, such as, enhanced DNA repair mechanisms but rather due to a multitude of other molecular targets, such as, membrane lipids. Platinum(II) complexes interact directly with lipids and induce changes in membrane phase behavior, that are dependent on PM lipid composition and other external factors. However, these interactions are complex and not fully understood. The design of novel platinum(II) chemotherapeutics should therefore account for these interactions and its consequences on platinum accumulation and efficacy in cells. Therefore, new modifications in the platinum(II) complexes should not only confer stability to reaction with other cell components (e.g., steric hindrance on the ZD0473) but also focus on permeation through resistant cells. The increased knowledge regarding cisplatin-lipid interactions should therefore contribute to a better understanding of the anti-tumor activity and how to overcome the mechanisms that determine resistance.

## Author Contributions

All authors listed have made a substantial, direct and intellectual contribution to the work, and approved it for publication.

## Conflict of Interest Statement

The authors declare that the research was conducted in the absence of any commercial or financial relationships that could be construed as a potential conflict of interest.

## Abbreviations

AFM: atomic force microscopy
aSMase: acid sphingomyelinase
DAPS: 1,2-diarachidonoyl-sn-glycero-3-phospho-L-serine
DDS: drug delivery systems
DISC: death-inducing signaling complex
DMPC: 1,2-dimyristoyl-sn-glycero-3-phosphocholine
DMPG: 1,2-dimyristoyl-sn-glycero-3-phospho-(1′-rac-glycerol)
DNA: deoxyribonucleic acid
DOPA: 1,2-dioleoyl-sn-glycero-3-phosphate
DOPC: 1,2-dioleoyl-sn-glycero-3-phosphocholine
DOPE: 1,2-dioleoyl-sn-glycero-3-phosphoethanolamine
DOPG: 1,2-dioleoyl-sn-glycero-3-phospho-(1′-rac-glycerol)
DOPS: 1,2-dioleoyl-sn-glycero-3-phospho-L-serine
DPH: 1,6-Diphenyl-1,3,5-hexatriene
DPPA: 1,2-dipalmitoyl-sn-glycero-3-phosphate
DPPC: 1,2-dipalmitoyl-sn-glycero-3-phosphocholine
DPPE: 1,2-dipalmitoyl-sn-glycero-3-phosphoethanolamine
DPPG: 1,2-dipalmitoyl-sn-glycero-3-phospho-(1′-rac-glycerol)
DPPS: 1,2-dipalmitoyl-sn-glycero-3-phospho-L-serine
DSC: differential scanning calorimetry
DSPG: 1,2-distearoyl-sn-glycero-3-phospho-(1′-rac-glycerol)
EPR: electron paramagnetic resonance
FADD: fas-associated protein with death domain FasL
GP: generalized polarization
HSPC: L-α-phosphatidylcholine, hydrogenated (Soy)
LUV: large unilamellar vesicle
MLV: multilamellar vesicle
NDDP: bis-neodecanoato-1,2-diaminocyclohexaneplatinum(II)
NHE1: Na^+^/H^+^ membrane exchanger-1
NMR: nuclear magnetic resonance
PC: phosphatidylcholine
PE: phosphatidylethanolamine
PG: phosphatidylglycerol
PM: plasma membrane
POPS: 1-palmitoyl-2-oleoyl-sn-glycero-3-phospho-L-serine
PS: phosphatidylserine
SM: sphingomyelin
TEER: *trans-*epithelial electrical resistance
